# The effect of tamoxifen and cisplatin on the disease-free and overall survival of patients with high risk malignant melanoma

**DOI:** 10.1054/bjoc.1999.1220

**Published:** 2000-06-02

**Authors:** E F McClay, M E T McClay, L Monroe, P L Baron, D J Cole, P H O'Brien, J S Metcalf, J C Maize

**Affiliations:** Department of Medicine, Division of Hematology/Oncology, and the Cancer Center, University of California, San Diego, 9500 Gilman Drive, La Jolla, CA, 92093-0063, USA; Departments of Medicine, Surgery and Dermatology and the Hollings Cancer Center, Medical University of South Carolina, 86 Jonathan Lucas Street, Charleston, SC, 29403-5851, USA

**Keywords:** tamoxifen, cisplatin, adjuvant therapy, melanoma

## Abstract

The adjuvant treatment of high-risk malignant melanoma remains problematic. Previously we reported moderate success in the treatment of metastatic disease using tamoxifen, cisplatin, dacarbazine and carmustine. Based upon data that suggested tamoxifen and cisplatin were the active agents in this regimen, we initiated a phase II trial of this combination in the adjuvant setting. We treated 153 patients with 4 cycles of tamoxifen (160 mg day^–1^, days 1–7) and cisplatin (100 mg m^–2^, day 2) for 28-day intervals. Patients received an anti-nausea regimen of dexamethasone with ondansetron or granisetron. During the first 2 years of follow-up, patients were evaluated every 2 months with a history, physical exam, laboratory work and computed tomography scans of the chest, abdomen and pelvis every 4 months. Thereafter, patients were evaluated every 3 months and radiographic studies were performed if necessary. Currently, with a median follow-up of 36 months, the disease-free survival (DFS) is 68.4% and overall survival (OS) is 84.5%. Kaplan–Meier analysis predicts a 5-year DFS of 62% with an OS of 79%. Relapses after 20 months have been rare. No effect of gender or number of positive lymph nodes was noted, however, stage of disease prior treatment was a factor. The major toxicity proved to be gastrointestinal in nature with nausea the most prevalent symptom. Minimal renal, haematologic and neurologic toxicity occurred. These preliminary results suggest that there is a positive impact of tamoxifen and cisplatin on both the DFS and OS of high-risk malignant melanoma patients. The 5-year projected DFS and OS compare favourably with those reported for the ECOG 1684 trial and warrant confirmation in a prospective randomized trial. © 2000 Cancer Research Campaign


British Journal of Cancer (2000) **83**(1), 16–21
© 2000 Cancer Research Campaign
doi: 10.1054/ bjoc.1999.1220, available online at http://www.idealibrary.com on IDEAL®

### The effect of tamoxifen and cisplatin on the disease-free and overall survival of patients with high risk malignant melanoma

**EF McClay[1], MET McClay[1], L Monroe[2], PL Baron[2], DJ Cole[2], PH O'Brien[2], JS Metcalf[2] and JC Maize[2]**

[1]Department of Medicine, Division of Hematology/Oncology, and the Cancer Center, University of California, San Diego, 9500 Gilman Drive, La Jolla, CA 92093-0063, USA; [2]Departments of Medicine, Surgery and Dermatology and the Hollings Cancer Center, Medical University of South Carolina, 86 Jonathan Lucas Street, Charleston, SC 29403-5851, USA

**Summary** The adjuvant treatment of high-risk malignant melanoma remains problematic. Previously we reported moderate success in the treatment of metastatic disease using tamoxifen, cisplatin, dacarbazine and carmustine. Based upon data that suggested tamoxifen and cisplatin were the active agents in this regimen, we initiated a phase II trial of this combination in the adjuvant setting. We treated 153 patients with 4 cycles of tamoxifen (160 mg day$^{-1}$, days 1–7) and cisplatin (100 mg m$^{-2}$, day 2) for 28-day intervals. Patients received an anti-nausea regimen of dexamethasone with ondansetron or granisetron. During the first 2 years of follow-up, patients were evaluated every 2 months with a history, physical exam, laboratory work and computed tomography scans of the chest, abdomen and pelvis every 4 months. Thereafter, patients were evaluated every 3 months and radiographic studies were performed if necessary. Currently, with a median follow-up of 36 months, the disease-free survival (DFS) is 68.4% and overall survival (OS) is 84.5%. Kaplan–Meier analysis predicts a 5-year DFS of 62% with an OS of 79%. Relapses after 20 months have been rare. No effect of gender or number of positive lymph nodes was noted, however, stage of disease prior treatment was a factor. The major toxicity proved to be gastrointestinal in nature with nausea the most prevalent symptom. Minimal renal, haematologic and neurologic toxicity occurred. These preliminary results suggest that there is a positive impact of tamoxifen and cisplatin on both the DFS and OS of high-risk malignant melanoma patients. The 5-year projected DFS and OS compare favourably with those reported for the ECOG 1684 trial and warrant confirmation in a prospective randomized trial. © 2000 Cancer Research Campaign

**Keywords:** tamoxifen; cisplatin; adjuvant therapy; melanoma

The current therapeutic options available for the treatment of patients with metastatic melanoma remain ineffective. Similarly, the search for new effective agents has not been successful identifying interesting compounds. Given the above, the search for successful adjuvant therapy for patients with high-risk melanoma has been hampered.

We and others have completed a number of studies employing what is referred to as the Dartmouth regimen, in the treatment of patients with metastatic disease (McClay et al, 1987, 1988, 1992*b*, 1993*c*; Richards et al, 1992; Saba et al, 1992; DelPrete et al, 1993; Lattanzi et al, 1993; Creagan et al, 1999; Saxman et al, 1999). To date, in 862 patients reported in the medical literature, treated with this regimen, the overall response rate is 31.8% (95% confidence limits 28.44–34.66%) with a complete response (CR) rate of 10%. These studies suggest a modest improvement in the overall and complete response rate with no statistically significant effect on DFS or OS. In the laboratory, we have identified a previously unrecognized synergistic cytotoxic interaction between TAM and DDP that may be the basis for the improved results observed with this regimen (McClay et al, 1992*a*, 1993*a*, 1993*b*, 1994). Based upon these clinical and laboratory data and the hypothesis that, if a clinically important synergistic interaction exists between TAM and DDP, then benefit would be most likely observed in the adjuvant setting, we began a phase II study of the combination of TAM and DDP in high-risk melanoma patients.

*Received 30 June 1999*
*Revised 16 November 1999*
*Accepted 8 December 1999*

*Correspondence to:* EF McClay

### PATIENTS AND METHODS

### Patient selection

In 1993, we began this phase II trial in cooperation with physicians in the primarily southeastern United States. A total of 39 community- and university-based physicians from the states of South Carolina, Georgia, North Carolina, Florida and Texas participated and treated patients on this programme. Patients eligible for this programme were required to have histologically documented malignant melanoma. Stage was determined by the use of the American Joint Commission on Cancer (AJCC) melanoma staging criteria. Selection factors for high-risk included those stage II patients who had a predicted risk of recurrence of at least 40% as determined by the tables of Clark et al (1989), as well as stage III and IV patients who could be rendered disease-free by surgical intervention. All pathologic slide review and interpretation of Clark's prognostic factors were conducted by one or both of the dermatopathologists (JSM, JCM) on the panel. Patients were required to have an ECOG performance status of 0–1 with normal renal, hepatic and haematologic function. Computerized tomograms (CT) of the chest, abdomen and pelvis without evidence of metastatic disease were required to be done within 1 month of the initiation of treatment. After informed consent, patients were started on treatment.

### Treatment

The treatment regimen included TAM 160 mg $day^{-1}$ on days 1–7 and DDP 100 mg $m^{-2}$ on day 2. DDP treatment was given in association with aggressive intravenous (i.v.) hydration and prophylactic anti-emetics. The anti-emetic regimen consisted of dexamethasone 20 mg i.v. in combination with either ondansetron (32 mg i.v.) or granisetron (10 μg $kg^{-1}$). Post-treatment prophylactic anti-emetics typically included metaclopromide (10 mg orally four times a day × 5 days) and ondansetron (8 mg orally twice a day × 5 days). The patients received a total of 4 cycles repeated at 28-day intervals.

### Evaluation

Following treatment, the patients were evaluated every 2 months for the first 2 years with a history and physical exam as well as laboratory monitoring. Similarly, CT scans of the chest, abdomen and pelvis were done every 4 months for the first 2 years. Thereafter the patients were evaluated by history, physical exam and laboratory measures only. The development of new symptoms prompted immediate radiographic investigation. The National Cancer Institute's New Common Toxicity Grading System was employed to grade all toxicity on this trial.

### Statistical measures

The disease-free survival (DFS) and overall survival (OS) were measured from the date of surgical intervention until recurrence of disease or death. The Kaplan–Meier method of survival analysis was employed to determine both DFS and OS (Kaplan and Meier 1958). The Mantel–Cox log-rank test was employed to determine statistical significance.

### RESULTS

### Patient characteristics

A total of 153 patients were entered onto this study, 92 males and 61 females with a median age of 51 years (range 19–78) (Table 1). All patients registered for the study are reported in this manuscript. All patients had an ECOG performance status of 0 with normal renal, hepatic and haematologic function. The number of patients at each stage of disease at entry onto the study was as follows: stage IIb – 64; stage III – 74; stage IV – 15 (Table 1). The median risk of recurrence for the stage IIb patients as predicted by the Clark's tables was 70% (Clark et al, 1989). This level of risk corresponds to that associated with a Breslow's depth of invasion of > 5 mm, if only depth is used to determine the risk of recurrence. Sites of disease for the stage IV patients prior to surgical resection included: skin (11), lung (2), brain (1), lymph nodes (3) and gastrointestinal (GI) (1).

### Survival

The median follow-up time for patients on this study 36 months. At the present time, the median DFS and OS have not yet been reached. The DFS is 68.4% while the OS is 84.5%. Kaplan–Meier analysis projected a 5-year DSF of 62% while the projected 5-year OS is 79% (Figures 1 and 2).

Cocconi et al (1992) reported a potential survival advantage for females treated on their programme which included TAM and DTIC. For this reason, we investigated the effect of gender on survival for our patient population. As can be seen in Figures 3 and 4, gender had no effect on either the DFS or OS in this trial.

It is well established that the number of lymph nodes that contain metastatic melanoma at diagnosis adversely effects the survival of patients with stage III disease. We investigated this effect in our patient population. We were unable to confirm an adverse effect on survival (Figures 5 and 6). However, the stage of disease of the patient prior to surgery did effect both the DFS and

**Table 1** Patient characteristics

| | Males | Females | Total |
|---|---|---|---|
| No. of patients | 92 | 61 | 153 |
| Stage IIb | 46 | 18 | 64 |
| Stage III | 42 | 32 | 74 |
| Stage IV | 4 | 11 | 15 |
| ECOG PS 0 | 92 | 61 | 153 |

Age: median, 50.9; range, 19–78.

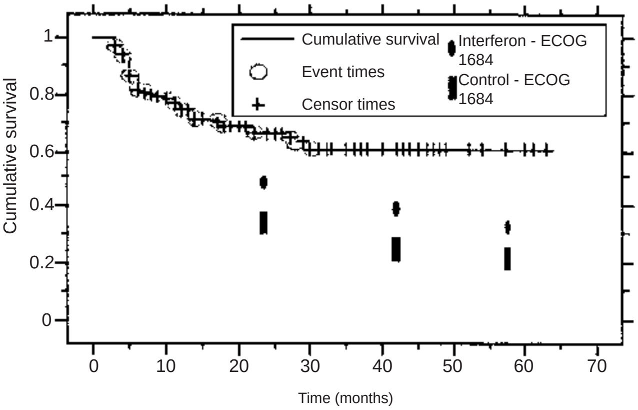

**Figure 1** Disease-free survival for all patients in comparison to the results of the ECOG 1684 Study

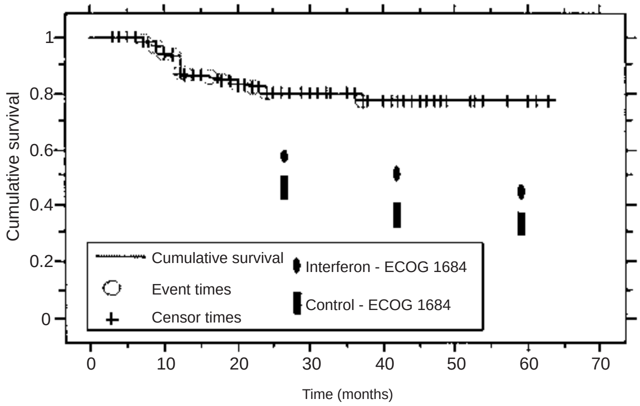

**Figure 2** Overall survival for all patients in comparison to the results of the ECOG 1684 study

*© 2000 Cancer Research Campaign*

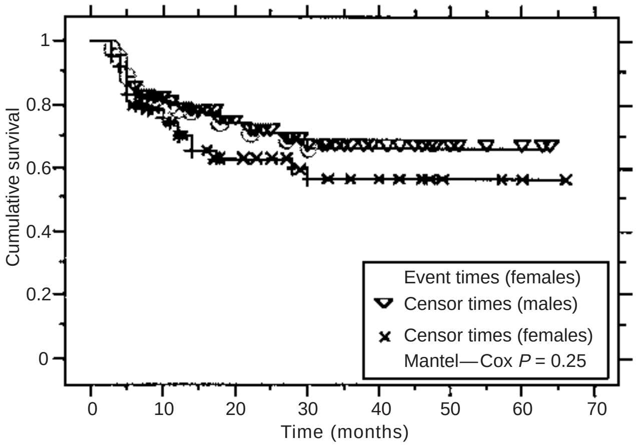

**Figure 3** Effect of gender on disease-free survival

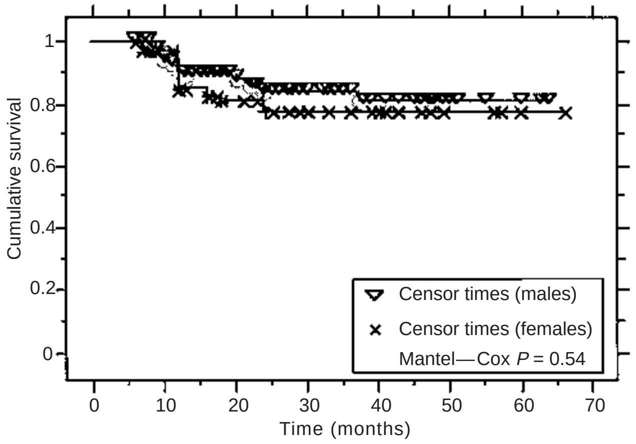

**Figure 4** Effect of gender on overall survival

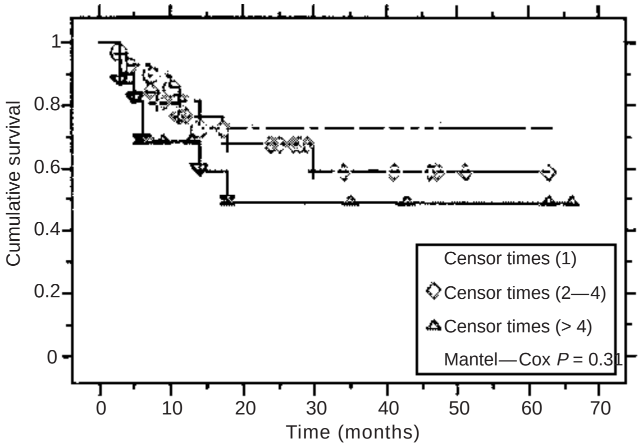

**Figure 5** Effect of lymph node status on disease-free survival

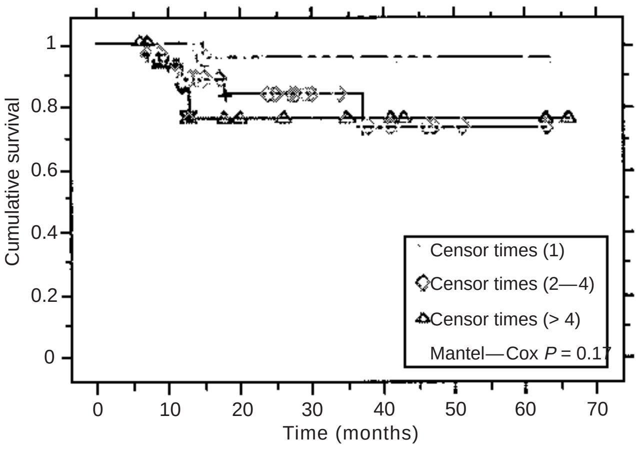

**Figure 6** Effect of lymph node status on overall survival

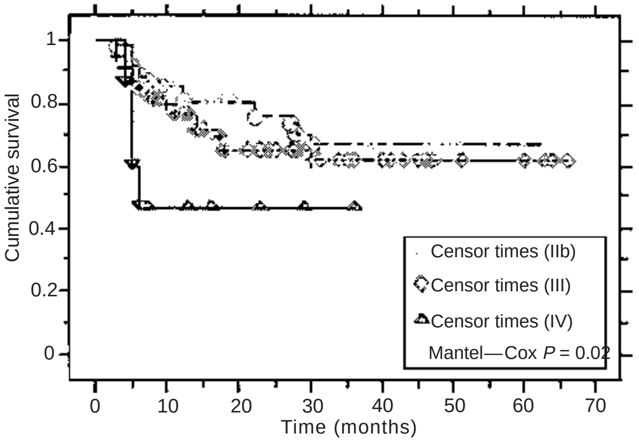

**Figure 7** Effect of stage on disease-free survival

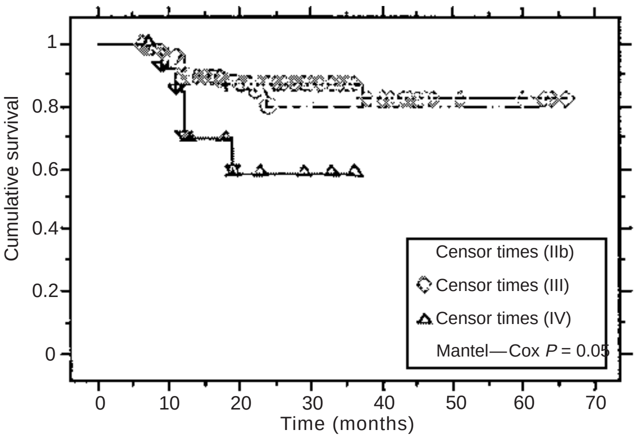

**Figure 8** Effect of stage on overall survival

OS experienced by patients treated on this programme (Figures 7 and 8). Those patients with stage IV disease prior to surgery had a statistically significant poorer DFS and OS in comparison to the stage II and III patients.

### Toxicity

Of the 153 patients treated on this regimen, 127 (83%) received all four planned treatments. Of the 26 (17%) who failed to complete

*© 2000 Cancer Research Campaign*

**Table 2** Toxicity

| Tox/Grade | 0 | I | II | III | IV |
|---|---|---|---|---|---|
| Renal | 120 | 32 | 1 | 0 | 0 |
| Nausea | 3 | 37 | 70 | 43 | – |
| Emesis | 24 | 10 | 86 | 0 | 33 |
| Neut/ Throm | 151 | 2 | 0 | 0 | 0 |
| Anaemia | 120 | 33 | 0 | 0 | 0 |
| Neuro | 148 | 5 | 0 | 0 | 0 |
| Ototoxicity | 151 | 2 | 0 | 0 | 0 |
| DVT/PE | 2/1 | | | | |

the programme, nine failed to do so because of the development of metastatic disease while on treatment while the others discontinued treatment due to toxicity. For the most part, persistent nausea with or without emesis was the most difficult symptom to treat (Table 2). Despite the use of prophylactic anti-emetics, nausea and emesis remained a significant problem for a majority of patients. Approximately 75% of all patients experienced grade II or higher nausea and/or emesis. While our numbers are too small to be significant, this problem appeared to be most significant in young women. In general, patients older than 60 years of age tolerated this regimen better from a GI standpoint than did the younger patients.

Only one patient developed grade II renal toxicity. This occurred in a patient with type II diabetes mellitus. Otherwise, minor elevations of the serum creatinine were observed in 32 additional patients, all of which returned to normal prior to the next treatment cycle. Further cycles were given with a 25% dose reduction of the DDP.

No significant haematologic, neurotoxicity or ototoxicity was encountered.

### DISCUSSION

Adjuvant therapy for patients with malignant melanoma has suffered from a lack of therapeutic agents that have activity in treating this disease. Despite this fact, a number of treatments have been evaluated in this setting, ranging from non-specific immune system stimulating agents to combination chemotherapy (Kirkwood et al, 1998). Levamisole, an antihelmintic agent with a variety of non-specific immune system effects was evaluated in several adjuvant melanoma studies, without clear success (Quirt et al, 1991; Spitler, 1991). Similarly, bacille Calmette-Guérin (BCG) has not been shown to effect either the DFS or OS (Czarnetzki et al, 1993).

The use of systemic chemotherapy has also not proved to be of benefit. Single-agent dacarbazine failed to improve the survival of high-risk stage I patients when compared with levamisole or placebo (Lejeune et al, 1988). Retsas et al (1995) have compared the survival of 87 stage III patients treated with adjuvant vindesine with the survival of 82 untreated patients in a non-randomized study. In contrast to other studies, the authors demonstrated a modest benefit in both DFS and OS in favour of the treated group. Not unexpectedly, combination chemotherapy has faired no better than other approaches (Pectasides et al, 1994).

Many recent studies have focused on the use of interferon (IFN) in the adjuvant setting with mixed results (Cascinelli et al, 1994; Creagan et al, 1995; Cole et al, 1996; Kirkwood et al, 1996; Pehamberger et al, 1998). To date only one study (ECOG 1684) has demonstrated a survival advantage for the use of high-dose IFN-α-2b (Kirkwood et al, 1996). This result was not confirmed in the yet to be published follow-up study (ECOG 1690) which found no survival advantage with the use of IFN-α-2b (http://cancertrials.nci.nih.gov/NCI_CANCER_TRIALS).

Our choice of the combination TAM and DDP stems from our clinical work with the Dartmouth regimen which suggests that TAM can overcome DDP resistance in selected patients with melanoma (McClay et al, 1987, 1989, 1992*b*, 1993*c*). In the early 1990s, in an attempt to determine the mechanism of action that might explain why this regimen might be effective, we began both clinical as well as laboratory investigations. We hypothesized that a previously unrecognized interaction between TAM and DDP was responsible for our observations. We subsequently conducted a clinical trial with this combination in previously untreated patients (McClay et al, 1993*c*). Patients were initially treated with DDP alone and, upon failure, subsequently treated with the combination of TAM/DDP. We observed a 33% response rate in patients treated with the combination after failure with single-agent DDP. While the clinical response of the patients was short lived, we believe that this response represented a biologically important observation, suggesting that clinical resistance to DDP could be overcome with high-dose TAM.

In the laboratory, we have confirmed the presence of a previously unrecognized synergistic cytotoxic interaction between TAM and DDP which may be the basis for the modest improvement in results that have been reported in several of the above studies (McClay et al, 1992*a*, 1993*a*, 1993*b*, 1994). In these experiments, TAM was able to make DDP-sensitive melanoma cells more sensitive and DDP-resistant cells, sensitive. Of interest, however, our in vitro data suggested that higher concentrations of TAM would be required to overcome DDP resistance (McClay et al, 1993*a*).

Based upon the above clinical and laboratory data and the rationale that the adjuvant setting provides the best opportunity to determine an effect of a treatment regimen on survival, we began this phase II pilot trial in 1993. Thirty-eight physicians, primarily from the south and southeastern United States, participated in the conduct of this trial. Melanoma was histologically confirmed and the risk of recurrence determined after review of the pathology slides by our reference dermatopathologists.

With a median follow-up time of 36 months, the median DFS and OS have not yet been reached. At present, the DFS is 68.4% and the OS is 84.5%. Kaplan–Meier analysis predicts a 5-year DFS of 62% with a 5-year OS of 79%. Relapses after 20 months have been rare and the survival curves appear to plateau after 25–30 months (Figures 1 and 2).

While others have suggested a survival advantage for female patients treated with TAM-containing regimens, we were unable to confirm this result (Cocconi et al, 1992). There was no statistically significant advantage observed for women treated with this regimen. Similarly, while there was a trend of worsening outcome (both DFS and OS) with an increasing number of positive lymph nodes at study entry, the trend did not reach statistical significance ($P = 0.26$).

In contrast, the stage of the patient at entry onto the trial did have a statistically significant effect on both DFS and OS. Those patients with stage IV disease prior to surgical intervention, had a poorer outcome as measured by both DFS and OS. Despite this, several of these patients have enjoyed clinically meaningful benefit in both DFS and OS.

© 2000 Cancer Research Campaign

This programme was reasonably well tolerated except for nausea and emesis. These symptoms were most common in patients who were younger than 60 years of age, especially young women. The reasons for this are unclear but may be related to the high doses of TAM employed in this regimen. Other significant toxicities were a rare occurrence. Two patients developed deep vein thrombosis, one of whom also suffered uncomplicated pulmonary emboli. This patient presented with a swollen leg and intermittent cough associated with mild dyspnoea following his last cycle of therapy. Multiple pulmonary emboli were found on ventilation/perfusion scan. The patient responded to anticoagulation without further symptoms.

How can we explain our results in light of the studies (Rusthoven et al, 1996) that have failed to clearly demonstrate a role for TAM in patients with metastatic disease? The key may be in the results of the in vitro studies using the combination of TAM/DDP in DDP-resistant cells (McClay et al, 1994). From a clinical perspective, most patients with metastatic melanoma are resistant to DDP. The overall response rate to single-agent DDP is in the range of 10–20% (Anderson et al, 1995). It follows therefore that, in the clinical setting, the majority of patients have melanoma cells that are de novo resistant to DDP. Assuming this is true, our in vitro data suggest that we should employ a higher dose of TAM when treating patients. This is the basis for the high dose of TAM that we have employed in this study. Other randomized and non-randomized studies have used a standard dose of TAM (20 mg day$^{-1}$).

A second obvious point is the fact that patients treated in the adjuvant setting have fewer malignant cells present than patients with measurable tumours. Thus, there is less risk that cells resistant to a particular therapy might be present in the patient. This was the basis for evaluating this combination in the adjuvant setting.

In summary, our data demonstrate that the use of the combination of TAM and DDP in high-risk melanoma patients results in an improvement in both DFS and OS in comparison to IFN-treated or untreated historical controls. It is stressed that these data are preliminary in nature and represent the first attempt employing this approach. We believe that these data support the conduct of a prospective randomized trial employing the combination of high-dose TAM and DDP to determine the effect of the combination on DFS and OS of patients with high-risk melanoma. This type of study can best be accomplished in the setting of a melanoma interest group or one of the national cooperative study groups.

### ACKNOWLEDGEMENTS

This study was supported in part by a grant from the National Cancer Institute CA52151 and the Bruce Banner Gorder Memorial Melanoma Fund. We acknowledge and thank the members of the Melanoma Interest Group for their participation in the care and treatment of patients on this program: Dr Gerald King, Dr Mark O'Rourke, Dr Jeffrey Giguere, Dr Kim Gococo, Dr Reginald J. Brooker, and Dr Jay Walls of the Cancer Center of the Carolinas; Dr Tripp G. Jones and Dr Rosemary Lambert-Falls of South Carolina Oncology; Dr Renwick Goldberg, Dr Lawrence Holt and Dr William Kellogg of Associated Medical Specialists and Dr James D. Bearden, Dr Eric Nelson and Dr James Bradof of Palmetto Hemotology Oncology, P.C. We also acknowledge Dr Mary Alice Ackerman, Dr Farouq Akbar, Dr Coleen Austin, Dr William Babcock, Dr Ayer Bala, Dr Elaine Beed, Dr Robert Bibb, Dr Elizabeth Christian, Dr Patti Dolan, Dr David Ellison, Dr Allan Freedman, Dr Bonnie Gearhart, Dr Ahmad Gill, Dr Ronald Goldberg, Dr Bernie Johnson, Dr Omar Kayaleh, Dr Gustav Magrinot, Dr Leland McElveen, Dr Michael Pavy, Dr George W. Schnetzer III, Dr Tom Seay, Dr Gary Thomas, Dr Robert Wall and Dr Joan Ween for their contributions.

### REFERENCES

Anderson C, Buzaid A and Legha S (1995) Systemic treatments for advanced cutaneous melanoma. *Oncology* **9**: 1149–1157

Cascinelli N, Bufalino R, Morabito A and Makie R (1994) Results of adjuvant interferon study in WHO melanoma programme. *Lancet* **343**: 913–914

Clark WH, Elder DE, Guerry DP, Braitman LE, Trock BJ, Schultz D, Synnestvedt M and Halpern AC (1989) Model predicting survival in stage I melanoma based on tumor proression. *J Natl Cancer Inst* **81**: 1893–1904

Cocconi G, Bella M, Calabresi F, Tonato M, Canaletti R, Boni C, Buzzi F, Ceci G, Corgna E and Costa P (1992) Treatment of metastatic malignant melanoma with dacarbazine plus tamoxifen. *N Engl J Med* **327**: 516–523

Cole BF, Gelber RD, Kirkwood JM, Goldhirsch A, Barylak E and Borden E (1996) Quality-of-Life-Adjusted Survival Analysis of Interferon Alfa-2a Adjuvant Treatment of High-Risk Resected Cutaneous Melanoma: An Eastern Cooperative Oncology Group Study. *J Clin Oncol* **14**: 2666–2673

Creagan ET, Dalton RJ, Ahmann DL, Jung SH, Morton RF, Langdon RM, Kugker J and Rodrigue LJ (1995) Randomized, surgical adjuvant clinical trial of recombinant Interferon alfa-2a in selected patients with malignant melanoma. *J Clin Oncol* **13**: 2776–2783

Creagan ET, Suman VJ, Dalton RJ, Pitot HC, Long HJ, Veeder MH, Vukov A, Rowland KM, Krook JE and Michalak JC (1999) Phase III clinical trial of the combination of cisplatin, dacarbazine, and carmustine with or without tamoxifen in patients with advanced malignant melanoma. *J Clin Oncol* **17**: 1884–1890

Czarnetzki BM, Macher E, Suciu S, Thomas D, Steerenberg PA and Rumke P (1993) Long-term adjuvant immunotherapy in stage I high risk malignant melanoma, comparing two BCG preparations versus non-treatment in a randomised multicentre study (EORTC Protocol 18781). *Eur J Cancer* **9**: 1237–1242

DelPrete SA, Maurer LH, O'Donnell J, Forcier FJ and LeMarbre P (1993) Combination chemotherapy with cisplatin, carmustine, dacarbazine and tamoxifen in metastatic melanoma. *Cancer Treat Rep* **68**: 1403–1405

Kaplan BL and Meier P (1958) Non-parametric estimation from incomplete observations. *J Am Stat Assoc* **53**: 457–461

Kirkwood J, Strawderman MH, Ernstoff MS, Smith TJ, Borden EC and Blum RH (1996) Interferon $\alpha$-2a adjuvant therapy of high-risk resected cutaneous melanoma: the Eastern Cooperative Oncology Group Trial EST 1684. *J Clin Oncol* **14**: 7–17

Kirkwood JM and Agarwala SS (1998) Adjuvant systemic therapy. In: *Cutaneous melanoma*, Balch CM, Houghton AN, Sorber AJ and Soong S (eds), p. 451. Quality Medical Publishing: St Louis, MO

Lattanzi SC, Tosteson T, Maurer LH, O'Donnell J, LeMarbre PJ, Del Prete SA, Forcier RJ and Ernstoff MS (1993) Dacarbazine (D), cisplatin (C), carmustine (B) +/– tamoxifen (T) in the treatment of patients (pts.) with metastatic melanoma: results of 5-year follow-up. *Proc ASCO* **390**: (Abstract)

Lejeune FJ, Macher E and Kleeberg U (1988) An assessement of DTIC versus levamisole or placebo in the treatment of high risk stage I patients under surgical removal of a primary melanoma of the skin. A phase II adjuvant study (EORTC protocol 18761). *Eur J Cancer* **24**: S81

McClay EF, Mastrangelo MJ, Bellet RE and Berd D (1987) Combination chemo/hormonal therapy in the treatment of malignant melanoma. *Cancer Treat Rep* **71**: 465–469

McClay EF, Sprandio JD, Mastrangelo MJ, Bellet RE and Berd D (1988) Importance of tamoxifen to a combination chemotherapy regimen for melanoma. *Proc ASCO* **7**: 250 (Abstract)

McClay EF, Mastrangelo MJ, Sprandio JD, Bellet RE and Berd D (1989) The importance of tamoxifen to a cisplatin containing regimen in the treatment of metastatic melanoma. *Cancer* **63**: 1292–1295

McClay EF, Christen R, Albright KA, Jones JA, Eastman A and Howell SB (1992*a*) Modulation of cisplatin resistance in human malignant melanoma cells. *Cancer Res* **52**: 6790–6796

McClay EF, Mastrangelo MJ, Berd D and Bellet RE (1992*b*) Effective combination chemo/hormonal therapy for malignant melanoma: experience with three consecutive trials. *Int J Cancer* **50**: 553–556

 © 2000 Cancer Research Campaign

McClay EF, Albright KA, Jones JA, Christen R and Howell SB (1993*a*) Tamoxifen modulation of cisplatin sensitivity in human malignant melanoma cells. *Cancer Res* **53**: 1571–1576

McClay EF, Albright KD, Jones JA, Christen RD and Howell SB (1993*b*) Tamoxifen modulation of cisplatin cytotoxicity in human malignancies. *Int J Cancer* **55**: 1018–1022

McClay EF, McClay ME, Albright KA, Jones JA, Christen R, Alcaraz J and Howell SB (1993*c*) Tamoxifen modulation of cisplatin resistance in patients with metastatic melanoma: a biologically important observation. *Cancer* **72**: 1914–1918

McClay EF, Albright KA, Jones JA, Christen RD and Howell SB (1994) Tamoxifen delays the in vitro development of resistance of cisplatin in human malignancies. *Br J Cancer* **70**: 449–452

Pectasides D, Alevizakos N, Bafaloukos D, Tzonou A, Asimakopoulos G, Varthalitis I, Dimitriadis M and Athanassiou A (1994) Adjuvant chemotherapy with dacarbazine, vindesine, and cisplatin in pathological stage II malignant melanoma. *Am J Clin Oncol* **17**: 55–59

Pehamberger H, Soyer P, Steiner A, Kofler R, Binder M, Mischer P, Pachinger W, Aubock P, Kerl H and Wolff K (1998) Adjuvant interferon α-2a treatment in resected primary stage II cutaneous melanoma. *J Clin Oncol* **16**: 1425–1429

Quirt IC, Shelley WE, Pater JL, Bodurtha AJ, McCulloch PB, McPherson TA, Paterson AHG, Prentice R, Silver HKB, Willan AR, Wilson K and Zee B (1991) Improved survival in patients with poor-prognosis malignant melanoma treated with adjuvant levamisole: a phase III study by the National Cancer Institute of Canada Clinical Trials Group. *J Clin Oncol* **9**: 729–735

Retsas S, Quigley M, Pectasides D, Macrae K and Henry K (1995) Clinical and histologic involvement of regional lymph nodes in malignant melanoma. *Cancer* **73**: 2119–2130

Richards JM, Gilewski TA, Ramming K, Mitchell B, Doane LL and Vogelzang NJ (1992) Effective chemotherapy for melanoma after treatment with interleukin-2. *Cancer* **69**: 427–429

Rusthoven JJ, Quirt IC, Iscoe NA, McCulloch PB, James KW, Lohmann RC, Jensen J, Burdette-Radoux S, Bodurtha AJ, Silver HKB, Verma S, Armitage GR, Zee B and Bennett K (1996) Randomized, double-blind, placebo-controlled trial comparing the response rates of carmustine, dacarbazine and cisplatin with and without tamoxifen in patients with metastatic melanoma. *J Clin Oncol* **14**: 2083–2090

Saba HI, Cruse CW, Wells KE, Klein CJ and Reintgen DS (1992) Treatment of stage IV malignant melanoma with dacarbazine, carmustine, cisplatin, and tamoxifen regimens: A University of South Florida and H. Lee Moffitt Melanoma Center study. *Ann Plast Surg* **28**: 65–69

Saxman SB, Meyers ML, Chapman PB, Destro AN, Panageas KS, Begg CB, Monaco F, Agarwala SS, Schuchter LM, Ernstoff MS, Einhorm LH, Kirkwood JM and Houghton AN (1999) A phase III multicenter randomized trial of DTIC, cisplatin, BCNU and tamoxifen versus DTIC alone in patients with metastatic melanoma. *Proc ASCO* **18**: 536a (Abstract)

Spitler LE (1991) A randomized trial of levamisole versus placebo as adjuvant therapy in malignant melanoma. *J Clin Oncol* **9**: 736–740

*© 2000 Cancer Research Campaign*
